# Can rights stop the wrongs? Exploring the connections between framings of sex workers’ rights and sexual and reproductive health

**DOI:** 10.1186/1472-698X-11-S3-S6

**Published:** 2011-12-16

**Authors:** Cheryl Overs, Kate Hawkins

**Affiliations:** 1 Michael Kirby Centre for Public Health and Human Rights, School of Public Health and Preventive Medicine Monash University, Alfred Hospital Campus Melbourne, Victoria 3004, Australia; 2 Institute of Development Studies, University of Sussex, Brighton BN1 9RE, UK

## Abstract

**Background:**

There is growing interest in the ways in which legal and human rights issues related to sex work affect sex workers’ vulnerability to HIV and abuses including human trafficking and sexual exploitation. International agencies, such as UNAIDS, have called for decriminalisation of sex work because the delivery of sexual and reproductive health services is affected by criminalisation and social exclusion as experienced by sex workers. The paper reflects on the connections in various actors’ framings between sex workers sexual and reproductive health and rights (SRHR) and the ways that international law is interpreted in policing and regulatory practices.

**Methods:**

The literature review that informs this paper was carried out by the authors in the course of their work within the Paulo Longo Research Initiative. The review covered academic and grey literature such as resources generated by sex worker rights activists, UN policy positions and print and online media. The argument in this paper has been developed reflectively through long term involvement with key actors in the field of sex workers’ rights.

**Results:**

International legislation characterises sex work in various ways which do not always accord with moves toward decriminalisation. Law, policy and regulation at national level and law enforcement vary between settings. The demands of sex worker rights activists do relate to sexual and reproductive health but they place greater emphasis on efforts to remove the structural barriers that limit sex workers’ ability to participate in society on an equal footing with other citizens.

**Discussion and conclusion:**

There is a tension between those who wish to uphold the rights of sex workers in order to reduce vulnerability to ill-health and those who insist that sex work is itself a violation of rights. This is reflected in contemporary narratives about sex workers’ rights and the ways in which different actors interpret human rights law. The creation of regulatory frameworks around sex work that support health, safety and freedom from abuse requires a better understanding of the broad scope of laws, policies and enforcement practices in different cultural contexts and economic settings, alongside reviews of UN policies and human rights conventions.

## Background

The phenomenon of sex workers waving red umbrellas and shouting slogans about human rights is now a fixture at national, regional and international conferences and meetings on sexual and reproductive health and HIV. Yet it is only recently that the international community, and particularly the UN, have seriously embraced sex worker rights activists’ logic about the connections between human and economic rights and public health goals.

*“In many countries*, *laws*, *policies and practices against sex workers limit their right to basic social economic rights such as access to education*, *health care*, *housing*, *banking facilities*, *inheritance*, *property and legal services. They may also lack of [sic] citizenship or legal status*, *resulting from migration or unfavourable regulations*.*” *[1]

Shifts in thinking about criminalisation of sex work have happened primarily because of the recognition that programmes that have been proven to reduce HIV such as condom distribution and STI services are being thwarted by laws against commercial sex and the broader legal framework that affect sex workers lives. Health considerations have driven support for reform of the laws around sex work within the highest echelons of the UN system. At the International AIDS Conference in Mexico in 2008 Ban Ki Moon, Secretary-General of the United Nations, made a speech in which he explained that,

“*In countries without laws to protect sex workers*, *drug users*, *and men who have sex with men*, *only a fraction of the population has access to prevention. Conversely*, *in countries with legal protection and the protection of human rights for these people*, *many more have access to services. As a result*, *there are fewer infections*, *less demand for antiretroviral treatment*, *and fewer deaths. Not only is it unethical not to protect these groups: it makes no sense from a health perspective.”*

In October 2009 the UN Human Rights Council adopted a resolution that urged States to eliminate laws that are counterproductive to HIV prevention, treatment and care including those that violate the rights of populations that are key to the dynamics of the epidemic and particularly affected by it. The UNAIDS Joint Outcome Framework of the same year made the removal of laws, policies and practices that block effective action on HIV a priority. It also mentioned sex work as part of a broader human rights agenda [2]. 2010 saw the launch of the Global Commission on HIV and the Law which has had a remit to review the links between human rights, HIV and sex work. Most recently at a UN sponsored regional consultation on HIV and sex work in the Asia Pacific region [3], Purnima Mane, UNFPA Deputy Executive Director, stated that:

*“Enabling sex workers to openly access prevention services with dignity must be part of every national HIV programme. Revising laws and policies and addressing attitudinal barriers will enhance the effectiveness of HIV prevention*, *improve access to health services*, *including reproductive health*, *and reduce violence against sex workers.” *[4]

That criminalisation of commercial sex limits access to health services is well documented. Anand Grover, the Special Rapporteur on the right of everyone to the enjoyment of the highest attainable standard of physical and mental health, comments:

*“Criminalization represents a barrier to accessing services*, *establishing therapeutic relationships and continuing treatment regimes*, *leading to poorer health outcomes for sex workers*, *as they may fear legal consequences or harassment and judgement. This is particularly concerning given that HIV has been noted to disproportionately affect sex workers in many regions*.*” *[5]

That this is especially the case for migrants, transgender or drug-using sex workers as well as people who sell sex in conflict and emergency situations is also well acknowledged [6]. Despite this understanding of the link between criminal law and ill-health international law; national regulatory frameworks and the pronouncements and demands of human rights activists and policy makers at international level may not yet line up in ways that lead to effective solutions for sex workers in practice.

## Methods

The literature review that informs this paper was carried out by the authors in the course of their work within the Paulo Longo Research Initiative (PLRI). The PLRI is a collaboration of scholars, policy analysts and sex workers. Its aim is to develop, consolidate and disseminate ethical, interdisciplinary information about sex work to improve the human rights, health and well-being of women, men and transgenders who sell sex. PLRI is a collaboration between the Global Network of Sex Work Projects, the Institute of Development Studies at the University of Sussex, the Michael Kirby Centre for Public Health and Human Rights at Monash University Medical School and the Centre for Advocacy on Stigma and Marginalisation (CASAM) in India.

The review covered academic and grey literature such as resources generated by sex worker rights activists, UN policy positions and print and online media. Sex workers networks’ email discussion groups, websites, blogs and other Internet communications were also tracked and analysed as they provide an important insight on the lived experience of sex workers in various different legal and cultural contexts.

The argument in this paper has been developed reflectively through extensive, long term research and advocacy involvement with key actors in the field of sex workers’ rights.

Burris et al. have stressed the challenge that researchers face in bridging the space between ‘law on the streets’ and ‘law on the books’ to achieve an accurate picture of the role of law in the health of marginalised groups [7]. By reviewing a variety of sources including; sex worker rights activist generated research and policy positions, academic literature, reports from UN agencies and non-governmental organisations (NGOs) and journalistic and online news we have been able to reflect on the connections and disconnections in various actors’ framings of sex workers’ sexual and reproductive health and rights.

This article considers resources related to (1) government policy and national law, (2) international frameworks and conventions and (3) sex worker rights declarations and advocacy documents. The positions outlined in international frameworks and sex worker rights declarations are reviewed over time to demonstrate developments in the framing of sex workers’ rights. We draw conclusions about the strengths and weaknesses in current framings of sex workers’ rights, based on an assessment of these sources and suggest future pathways for a rights based approach to sex work and the law particularly as it pertains to sexual and reproductive health and the role of governments and public health organisations.

## Results and discussion

### Governments and national laws

Historically, criminal law has addressed sex work through criminalising the selling of sexual services, with the imposition of penalties upon sex workers themselves and through criminalisation of various practices around sex work. These include keeping a brothel; recruiting for or arranging the prostitution of others; living off the proceeds of sex work; solicitation; and facilitating sex work through the provision of information or assistance. Although selling sex is not directly criminalised in many countries worldwide, there are widespread reports that sex workers are nonetheless treated as criminals where activities around sex work itself are criminalised, or through the use of pre-existing laws (not specific to sex work) to harass, intimidate or justify the use of force against sex workers [8]. In some settings, such as Sweden, the criminalisation of the clients of sex workers has been employed.

Some of the laws and policies that are designed to respond to matters unrelated to commercial sex have a legitimate purpose such as keeping public roads and pathways clear or protecting people from nuisance. But many are other discriminatory laws such as those against homosexuality and transgenderism. Transgender and male sex workers are often subject to laws against homosexuality or obscenity [9]. Drug-using sex workers in particular are subject both to laws about those activities and brutal enforcement [10]. Vagrancy laws are notoriously used to criminalise sex workers. For example, street-based sex workers in Malawi are arrested on the charge of “Rogue and Vagabond” a colonial-era legal term for what is more commonly known as loitering [11].

Executive orders and subordinate legislation (of varying degrees of legitimacy) often set out where and how the sex industry can operate. Such orders are often associated with ‘crackdowns’ and other forms of suppression that extend beyond the technical limit of the law. In 2010, all sex workers were ordered to leave the Nigerian capital Abuja by such an order [12]. However executive decrees may be also associated with toleration. For example, police may be instructed to ignore the law in relation to brothels in exchange for compliance with HIV prevention activities such as supplying condoms or ensuring that no HIV positive women sell sex. Executive orders were a central lever to secure the compliance of brothel keepers in Asia, such as in Cambodia, to comply with 100% Condom Use programmes [13].

In wealthier countries sex workers may be ordered by law to attend mandatory counselling or rehabilitation or subject to quasi-official “naming and shaming” by police and vigilantes. For example many US police forces publish pictures of sex workers , particularly those living with HIV [14] and the London Police issued Anti-Social Behaviour Orders to six street sex workers in London and published their full names, photos and dates of birth on their website because they were “persistent offenders” [15]. Sex workers and others associated with sex work are banned from entering several countries including Japan and the United States of America.

Increasingly offences related to sex work have been conflated in various combinations into the concept of “sex trafficking” or “trafficking and sexual exploitation” and given legislative expression, including in laws that criminalise buying sex. These can also stem from efforts to combat violence against women. Commercial sex may also be regulated and licensed, and these schemes take different forms. Some schemes permit commercial sex in certain areas in streets or in brothels, other schemes permit commercial sex that accords with prescribed standards, such as the licensing of managers and the registration of workers.

An increasingly prominent legal mechanism for addressing sex work is policies and directives that aim to reduce sex work, exploitation and human trafficking by removing women and children from brothels and other places in which they are deemed to be victims of trafficking and sexual exploitation. Sex workers have called this “Raid and Rescue” or “Rescue and Rehabilitation” and implicated it in serious human rights abuses against nationals and migrant sex workers [16]. In Cambodia, Human Rights Watch identified arbitrary detention in government rehabilitation centres as a result of the enforcement of the Law on the Suppression of Human Trafficking and Sexual Exploitation as a threat to both human rights and health. Human Rights Watch describe how detainees reported rape and missed medication among other consequences of their detention [17]. Sex workers report a variety of abuses including; rape by guards and other prisoners, beatings, deaths in custody, lack of medication or medical care (notably for HIV positive, mentally ill and pregnant inmates), abuse of disabled people, lack of hygiene facilities and toilets and food and water. Writing about raids by a Christian NGO in Sangli, India Rao and Sluggett explain,

“*At each stage of the intervention*, *women experience gross human rights violations. Their right to livelihood and their right to reside wherever they want is suspended; their right to privacy and their right to liberty and equality before the law is taken away because they are being ‘taken care of’. Some sex workers sustain injuries in trying to escape being ‘rescued’.” *[16]

Community violence against sex workers, as the public take “law enforcement” into their own hands, is also an issue of concern. For example, this year women in Nigeria threatened to create a “squad to apprehend and possibly prosecute call girls” who had moved to their area after the crackdown mentioned above [18] In India a mob burnt down a guest house in Naroda (Gujarat, India) reportedly to “teach a lesson” to criminals involved in law-breaking. Two women were injured and one died [19]. In Bolivia in 2007, sex workers were ejected from their homes by angry crowds who stormed the “red light” area and attacked them in protest at the sex industry. As a result sex workers refused to co-operate with the regulations that require them to submit to medical examinations if they are to work legally. Ten sex workers sewed their lips together and others fasted in a clinic demanding that the mayor reopen the area and secure their safety [20]. Negotiation with legislator Guillermo Mendoza led to the suspension of the protest [21]. Indeed, serial murderers of sex workers have sometimes invoked the anti-sex work aim of the law by claiming to be ridding society of such women. The ‘Green River Killer’ of sex workers in the US said to police "I thought I was doing you guys a favor, killing prostitutes… you guys can't control them, but I can" [22]. The Yorkshire Ripper in the UK said “The women I killed were filth, bastard prostitutes who were just standing round littering the streets. I was just cleaning the place up a bit.” [23]

While health research concentrates on the important task of identifying levels of HIV among sex workers and measuring the impact of HIV interventions, much less is known about how law influences the sex workers’ opportunities to avoid sexually transmitted infections, violence, unwanted pregnancies and access to sexual and reproductive health services and dual protection. Yet policy positions and statements from sex worker rights activists point to the intersections of sexual and reproductive health and law and their human rights.

### What do sex workers rights statements say?

An analysis of key position statements from sex worker rights groups on their rights shows that a concern with health and wellbeing is related to a host of rights claims that relate to the structural barriers – the socioeconomic, cultural and political factors – that limit sex workers’ ability to participate in society on an equal footing with other citizens. The International Network of Sex Work Projects (NSWP) has long argued that the criminalisation of commercial sex inhibits sex workers’ ability to realise their sexual, reproductive and other rights by rendering them vulnerable to exploitation, invisibilising abuses [24]; limiting access to health services and information and safe places to work [25] and denying them the economic, cultural and social rights conferred by citizenship. They argue that laws that try to abolish or limit sex work both limit access to services and create the unsafe working conditions that drive transmission of sexually transmitted infections, HIV, unwanted pregnancy and other threats to health. This happens, the NSWP says, because sex workers, especially women, in many settings face a choice between risking arrest and violence by working independently and working in venues that offer some degree of protection but which may be exploitative or abusive because they are operated beyond the reach of law and often by criminals.

At the same time sex worker rights activists have spoken about the effects of unfavourable laws that reach beyond health issues. They argue that states’ protection of human rights is a matter of justice, not simply an instrument for reducing sex workers’ role as vectors of disease. Networks of sex worker rights activists have vigorously claimed a space to link sex worker empowerment and health issues with human rights. Slogans such as “sex workers rights are human rights” [26] and “only rights can stop the wrongs” [27] capture the idea among activists that better health outcomes are a consequence of improved human rights.

Contemporary sex workers’ positions on human rights were first documented in the World Charter for Prostitutes' Rights which was drafted as a result of the World Whores Congress of 1985 [28]. The Charter identifies stigma and discrimination as a key barrier to the realisation of rights. It has a strong focus on human rights from a feminist perspective and claims that sex work is an occupation that should be legal for sex workers, their employers, their clients and families. It calls for the decriminalisation of sex work and distinguishes between sex work and sexual abuse. It demands the regularisation of working conditions in the sex industry in line with other comparable businesses and calls for the creation of social services for sex workers. It opposes discriminatory mandatory testing but calls for universal access to sexual and reproductive health care (interestingly it does not mention HIV although the virus had been recently identified. This appears to be due to fear of stigma).

The Sex Workers' Manifesto from the First National Conference of Sex Workers in India, 1997 [29] echoes the Charter in its call for an understanding of sex work as a legitimate occupation. The Manifesto also explores how patriarchy and morality intersect in understandings of sexuality. It rejects the idea of sex as simply an instrument of reproduction which, in their analysis, leads to compulsory heterosexuality which curbs women’s sexual freedom. It points out that such narrowly focused understandings of women’s sexuality easily lead to the splitting of women into “madonnas and whores”. Instead it calls for autonomous sexuality and mutually pleasurable safe sex, where people have the right to consent and sex is free of guilt and oppression.

This manifesto marks the important entry of sex workers from a poor country into a global movement that had previously focused on legal and policy issues as they apply in Europe and North America. Unlike the Charter, the Indian declaration challenges the role of choice in framing sex work as an occupation, “when do us women have access to choice within or outside the family? Do we become casual domestic labourer (sic) willingly? Do we have a choice about who we want to marry and when? The choice is rarely real for most women, particularly poor women”. Its analysis places sex work in the context of a social and economic system where options for women are limited and where access to income generating activities of any sort is prized. Within this framework sex work is not necessarily seen as the worst option for women. The Manifesto rejects charitable interventions, rescue and rehabilitation, which it sees as the typical tools of those who seek to abolish the sex industry, and instead calls for the respect of human rights and improvement of sex industry standards.

The Taipei Declaration of Sex Workers’ Human Rights was written at the 1998 World Action for Sex Workers Rights in response to a crackdown that revoked the licenses of Taipei sex workers [30]. Although a short document it is interesting in its use of reference to the Beijing Plan of Action and the Report of the UN Special Rapporteur on Violence Against Women in order to counter the city government’s abolitionist interpretation of sex work as supported by international law on trafficking.

The Declaration of the Rights of Sex Workers in Europe was created in 2005 in response to what sex workers perceived to be a swing toward measures to constrain sex industries across Europe in order to safeguard public health and/or limit human trafficking [31]. It identifies human, labour and migrants rights that sex workers should be entitled to under international law as citizens and outlines specific measures that states should take to fulfil these rights. It does not call for special rights for sex workers but rather argues that to make sex work safe people engaged in the sale of sexual services should not be denied human rights. It hinges on the non-discrimination clause found in most human rights treaties.

The Declaration of the Rights of Sex Workers in Europe covers many of the topics that are explored in earlier statements on sex workers’ rights including health, freedom from slavery and exploitation and associated labour rights. It also provides more detail on the right to seek asylum and to non-refoulement – protection from forced return to places where their lives or freedoms are threatened – reflecting the document’s concern with migrant workers. The right to marry and found a family is also identified as an area of concern. The document calls upon governments to ensure that engagement in sex work does not restrict the ability of sex workers to obtain other employment or to marry, or lead to discrimination against sex workers’ families by public authorities, “Current or former engagement in sex work should not be considered grounds for challenging a person’s fitness to be a parent or have custody of his or her children”. ([31]. p9)

The most recent call for sex workers rights which is considered by this paper is the Pattaya Draft Declaration on Sex Work in Asia and the Pacific (2010) (the Pattaya Draft Declaration) [32]. This Declaration was agreed by sex workers representing the Asia Pacific Network of Sex Workers and organisations and NGOs from the region. This draft is currently under consultation with the Asia Pacific Network of Sex Workers. This declaration is framed as a “unified and rights based approach to the reduction of HIV among adult sex workers” ([32]. p1). Unsurprisingly, access to services is a key theme – particularly efforts to counter stigma and discrimination on the grounds of occupation or HIV status. The document also calls for expanded economic and social opportunities; better health care for male and transgender sex workers, an end to the pressures for abortion and sterilisation, and protection from discrimination for sex workers of all genders living with HIV.

In contrast to some of the earlier statements, the Pattaya Draft Declaration focuses on all genders and does not refer to women’s rights or have an overtly feminist analysis of oppression. Rather the unifying argument of the piece is the importance of the inclusion and the meaningful and active involvement of sex workers in decision making, programming, advocacy and action to address stigma and discrimination and capacity building.

*“The starting point for a rights based approach to HIV and sex work is the formation of a partnership in which sex workers’ contributions to policy and programme development is encouraged*, *supported*, *recognised and valued. This cannot occur in coercive environments…or where sex work is governed by laws that address trafficking” *([32]. p1).

Coercive efforts to control or reduce sex work identified by the document include mandatory medical treatment or procedures, raids, forced rehabilitation and programmes implemented by police or based upon detention of sex workers. The document states that in some circumstances these may constitute torture and other cruel, inhuman or degrading treatment or punishment.

What links all of these documents is a call for sex work to be recognised as a legal occupation to pave the way for commercial sex to be conducted in safe workplaces, for violence to be reduced and to enable universal access to appropriate health services. According to the sex workers’ rights movement, removing criminal laws against sex work could provide an opportunity to reconfigure the way in which sex workers’ sexual and reproductive rights can be realised. Where sex work is recognised as an occupation, SRHR could become a matter of labour, rather than criminal law. This would be linked to health and safety in the workplaces and environments within which the sale of sex takes place. In settings where they are recognised, the trade union movement could be harnessed to enforce positive norms and standards. In settings that are less conducive to unionisation – or where this is not a popular form of organising around labour issues – business regulations and workplace health regulations would still provide protection. Furthermore the recognition of sex work as an occupation would enable sex workers to access state and privately provided services and benefits such as education, pensions, welfare payment, healthcare and equal access to infrastructure such as housing, sanitation and transport.

Sex worker rights activist statements and policy positions make the argument that good health depends on sex workers having the same rights that protect other people in their roles as workers, business people, family members, tenants, patients, taxpayers etc. On this analysis it is not logical to aim to promote health in isolation from addressing the drivers of vulnerability through human rights.

### The international legal framework and its interpretation in national constitutions, bills of rights and legal decisions

A plethora of international instruments addresses prostitution, trafficking and technical and medical ethical issues related to sex work. The Convention for the Suppression of the Traffic in Persons and the Exploitation of the Prostitution of Others (the Convention) was adopted in 1949 [33]. It grew out of “social purity” campaigns which were preceded by women’s international organising around the “white slave trade” [34,35]. The Convention describes prostitution as “incompatible with the dignity and worth of the human person and endanger(ing) the welfare of the individual, the family and the community” ([33]. p1). It frames prostitutes as “victims” and “fallen women” as tricked or exploited persons [33].

The Protocol to Prevent, Suppress and Punish Trafficking in Persons, Especially Women and Children, supplementing the United Nations Convention against Transnational Organised Crime, of 2000 [36], defines trafficking as

*“the recruitment*, *transportation*, *transfer*, *harbouring or receipt of persons*, *by means of the threat or use of force or other forms of coercion*, *of abduction*, *of fraud*, *of deception*, *of the abuse of power or of a position of vulnerability or of the giving or receiving of payments or benefits to achieve the consent of a person having control over another person*, *for the purpose of exploitation.”* [p1]

Exploitation is further defined to include the prostitution of others or other forms of sexual exploitation. Additionally, the Protocol states that the consent of any victim of trafficking is deemed irrelevant where circumstances such as vulnerability or abuse of power exist.

Article 6 of the 1979 Convention on the Elimination of Discrimination Against Women (CEDAW) [37] calls on states to suppress “all forms of traffic in women and exploitation of prostitution of women.” It does not require States to suppress consensual, adult sex work but calls for the suppression of “all forms of traffic in women and exploitation of prostitution of women”. The term “exploitation of prostitution” has not been defined within the Convention; Article 6 is read to mean that prostitution is discriminatory in its impact on women and as such is an abuse under the premise of non-discrimination. However, Doezema [34] has argued that the inclusion of the word “exploitation” implies that the convention does not consider all prostitution inherently coercive and uses evidence from the negotiations around its drafting to support this point. Interpretation of the “exploitation of prostitution” is important as it goes to the heart of whether CEDAW considers prostitution itself exploitative or whether prostitution is a system and practice within which human rights abuses of women are prevalent.

The General Recommendation of the Committee on the Elimination of Discrimination against Women, 1993 [38] frames sex work as a practice that contributes to shaping the traditional attitudes that devalue women to help justify gender based violence as well as being a form of gender-based violence in and of itself. It continues to mirror the language of CEDAW in its use of the term the “exploitation” of prostitution. This led Balos [39] to interpret CEDAW as positing prostitution as a violation and inconsistent with human rights regardless of whether it is consenting.

The Declaration on the Elimination of Violence Against Women (DEVAW), 1993, uses the term “forced prostitution” to refer to the “type of physical, sexual and psychological violence” that is “a violation of the rights and fundamental freedoms of women and impairs or nullifies their enjoyment of those rights and freedoms” [40]. This represents a shift in understanding from “prostitution” per se or “the exploitation of prostitution” to “forced prostitution” as a human rights abuse. By making a distinction between voluntary and forced prostitution the Declaration could be seen as a tacit admission that not all prostitution is an abuse of women’s rights. This language was later used in the Beijing Platform of Action [41].

Lack of clarification of these terms is a key obstacle to State responses to adult sex work that protect health and human rights. As Grover points out, terms such as “vulnerability” and “abuse of power” remain undefined within the Protocol [5]. Indeed they have no independent legal meaning, and nor do concepts such as “dignity” and “exploitation.”

Other conventions may contain provisions that can support efforts to reduce sex workers’ vulnerability and improve sexual and reproductive health. These include the International Covenant on Economic, Social and Cultural Rights which protects the right to freely chosen, gainful work. The International Convention on the Protection of the Rights of All Migrant Workers and Members of Their Families applies to a significant number of sex workers who travel between States to engage in sex work [5].

In practice, the patchwork of ideas contained within these international instruments often manifest themselves as laws such as the Cambodian Law on the Suppression of Human Trafficking and Sexual Exploitation the enforcement of which was discussed earlier in this paper. The law deems all women who join the sex industry voluntarily to be victims of sexual exploitation and they have the same status as women who have been subject to kidnapping, rape, imprisonment and other crimes committed to force them to sell sex against their will. The legislation is aimed at preventing human trafficking and sexual exploitation, but the law actually prohibits all activities around sex work and effectively criminalises the sex sector in its entirety.

In some cases human rights as embodied in national constitutions, bills of rights and international conventions underpin legal decisions that have led to less punitive approaches to sex work. Although they remain rare they may provide an important signpost to resolving sex workers’ human rights claims. A Canadian court recently accepted a constitutional challenge to three provisions of Canadian sex work law: keeping a common bawdy house, or brothel, communicating for the purposes of prostitution and living on the avails of the trade. The judge in the case ruled that even though the act of prostitution is not illegal in Canada, Parliament has seen fit to criminalise most aspects of prostitution and that these provisions violated sex workers’ constitutional right by forcing sex workers to choose between their liberty interest and their right to security of person as protected under the Canadian Charter of Rights and Freedoms.

Some changes in legislation that recognise sex work as legitimate labour, and to safeguard sex workers from abuse, come from wealthy countries including the Netherlands, Germany, Australia and New Zealand. A recent evaluation of the reform in New Zealand showed that it has enabled people working in the sex industry to access protections through labour and anti-discrimination laws and that they are more able to access health services [42].

There have been similar developments in both legislatures and courts in developing and middle-income countries. In Brazil, sex work has been added to an official list of occupations that enable sex workers to claim social benefits. In South Africa, an industrial tribunal initially rejected a sex worker’s right to bring a case for unfair dismissal against an illegal brothel on the basis that an employment contract between a prostitute and her employer cannot be recognised or enforced because it is unlawful and contrary to public policy. However an appeals court held that although the contract was unlawful, an employment relationship existed between them so the sex worker’s claim fell within the scope of the Labour Relations Law [43]. Similarly national constitutions have been invoked in courts in Bangladesh [44] and Colombia [45] which have recently found in favour of recognising sex work as an occupation, clearing the way for sex workers to vote, pay tax and claim other civil rights.

In Taiwan, commitment to bring legislation into harmony with the International Covenant on Civil and Political Rights and the International Covenant on Economic, Social and Cultural Rights resulted in anti-sex work law being abolished in 2009 on the grounds that punishing commercial sexual transactions forces sex workers underground leaving them open to abuse. The government stated that while sexual transactions between consenting adults should be governed by personal, educational and religious considerations, rather than by laws, the sex trade should be regulated like any other occupation. Subsequent to this, the Constitutional Court declared the existing legislation unconstitutional, and ordered that it cease to be in effect within 2 years [46].

## Discussion and conclusion

Debates on how best to govern commercial sex and reduce its potential to generate health problems have a long and complex history out of which little consensus has emerged. These debates are often tied up with narratives about disease control and harm reduction; female virtue and empowerment; and migration and slavery [47-49]. In recent years attention to sex workers’ sexual and reproductive health and rights has been prompted by a desire to control and reduce HIV. However, political framings of sex work within a paradigm of exploitation and slavery have also gained enormous political traction. In this paradigm the sex industry is imagined to be inherently abusive and those involved in it lacking agency. In this way sex work is conflated with “sex trafficking”, which has the effect of creating a powerful force against public health or other initiatives aimed at making sex work safer lest it be considered as colluding with exploitation or even slavery.

Although some of those rights talked about by sex worker rights activists are mentioned in conventions, some are undermined or weakened by international law whose jurisprudence has traditionally constructed sex work as an affront to human dignity. The danger of a continuation of the status quo is that formal rights frameworks become even less relevant to sex workers. There is a significant tension between those who wish to uphold the rights of sex workers in order to reduce vulnerability to ill-health and those who insist that sex work is itself a violation of rights and human dignity and cannot therefore be regarded by law as a legitimate occupation.

There is much in the international human rights conventions and law that accords with sex worker rights activists’ demands. However our analysis of international legislation and national legal responses highlights instances of difference and contestation. In some cases sex worker rights claims push up against the boundaries of conventional interpretations of human rights. Confused definitions and understandings of sex work, sexual exploitation and human trafficking are not merely a historical artefact. They are “live” issues that require informed debate and resolution if the UN and international law are to play a positive role in improving the health and human rights of women, transgenders and men who sell sex.

Sex worker rights activists clearly have reason to be delighted about renewed attention to the idea that punitive laws against sex work should be removed. However the instrumentalist argument made by some public health professionals that sex workers’ rights are necessary in order to ensure that sex workers’ access HIV prevention services is quite different to the framing of sex workers’ rights in the various declarations and position papers from activists that we reviewed. While both health authorities and sex workers stress the importance of access to HIV and STI testing and condoms as the key to good health outcomes, sex worker rights activists place equal or more weight on their ability to work in safe, clean and fair workplaces.

Simply removing criminal law is unlikely to be a solution in itself. Good regulations, effective health programmes and equitable policy do not automatically begin when criminal laws are removed even in rich and well governed countries, let alone where regulatory systems generally are not well organised. If criminal laws are replaced with discriminatory or inappropriate policy and law the health and safety conditions in the sex industry could actually be made worse and access to services denuded.

There are some clear gaps in the discourse around sex work. Most literature focuses on female sex workers and much of it on young women. Far less attention is paid to the health issues facing male, transgender, migrant, drug using or older sex workers. Better understanding of the broad scope of laws policy and enforcement practices that affect sex workers in the many very different cultural contexts and economic settings is needed urgently. The lived experience of sex workers is rarely the topic of ethical, methodologically rigorous examination and there is little systematic collection of data about law and its impact.

Careful review of UN policy and the international human rights conventions and related law is much needed too. Such a review should begin with recognition that the international legal framework is sometimes at odds with grounded understandings of entitlements and frequently at odds with itself. Sex workers’ broad spectrum of claims is based on collective understandings of human rights grounded in justice rather than on sophisticated technical knowledge of international human rights conventions and laws and the various roles of the institutions and individuals upon whom they confer rights and duties. It is possible to create regulatory frameworks for commercial sex that support safe practice by recognising commercial sex as a legitimate activity. Only in this way does it become possible for sex workers to be accorded all of their human rights.

To develop a formula for law, policy and enforcement practices that enables a safer sex industry, supports sex workers to claim human rights and protects health including reducing vulnerability to HIV, governments require three things:

1. Precise language to describe the regulatory environments for commercial sex, the consequences of possible regulatory options, and the potential solutions;

2. Accurate local data about the domestic laws, policies and enforcement practices to be remedied, and their impact, intended and unintended, on the health of male, female and transgender sex workers; and

3. A commitment to human rights standards and norms, including the right to work, to equal protection under the law, to freedom of association, and a recognition of the right of consenting adults to form sexual relationships of their choice provided others are not harmed.

Health projects are well placed to gather information about law and its impact in much of the world. Those with policy and advocacy capacity can actively support sex workers’ campaigns which are notoriously under-resourced. The best course for health agencies is to listen to sex workers and join with them to demand the fundamental changes locally, nationally and internationally that can make sex work safe, enable sex workers to realise their human rights and enable access to the sexual and reproductive services they need to stay healthy.

## List of abbreviations used

AIDS: Acquired Immune Deficiency Syndrome; CASAM: Center for Advocacy on Stigma and Marginalisation; CEDAW: Convention on the Elimination of Discrimination Against Women; DEVAW: Declaration on the Elimination of Violence Against Women; HIV: Human Immunodeficiency Virus; NGO: Non Governmental Organisation; NSWP: International Network of Sex Work Projects; PLRI: Paulo Longo Research Initiative; SRHR: Sexual and Reproductive Health and Rights; STI: Sexually Transmitted Infection; UN: United Nations; UNAIDS: Joint United Nations Programme on HIV/AIDS; UNFPA: United Nations Population Fund; US: United States.

## Competing interests

The authors declare that they have no competing interests.

## Authors’ contributions

Both authors were involved in drafting the paper and contributed to the final analysis and recommendations.
